# iTRAQ-Based Comparative Proteomics Analysis of Urolithiasis Rats Induced by Ethylene Glycol

**DOI:** 10.1155/2020/6137947

**Published:** 2020-05-15

**Authors:** Yanan Cao, Bin Duan, Xiaowei Gao, E. Wang, Zhitao Dong

**Affiliations:** ^1^Department of Anesthesiology, Xiangya Hospital Central South University, Xiangya Road 87#, Changsha, Hunan 410008, China; ^2^Department of Urology, Second Xiangya Hospital, Central South University, Changsha, Hunan, China 410011., China

## Abstract

Nephrolithiasis is a frequent chronic urological condition with a high prevalence and recurrence rate. Proteomics studies on urolithiasis rat models are highly important in characterizing the pathophysiology of kidney stones and identifying potential approaches for preventing and treating kidney stones. The isobaric tags for relative and absolute quantification (iTRAQ) were performed to identify differentially expressed proteins (DEPs) in the kidney between urolithiasis rats and control rats. The results showed that 127 DEPs (85 upregulated and 42 downregulated) were identified in urolithiasis and control rats. The functions of DEPs were predicted by Gene Ontology (GO) analysis, Kyoto Encyclopedia of Genes and Genomes (KEGG) enrichment analysis, and protein–protein interaction (PPI) network analysis. The expression of four upregulated proteins (Tagln, Akr1c9, Spp1, and Fbn1) and four downregulated proteins (Hbb, Epb42, Hmgcs2, and Ca1) were validated by parallel reaction monitoring (PRM). Proteomics studies of ethylene glycol-induced urolithiasis rat models using iTRAQ and PRM helped to elucidate the molecular mechanism governing nephrolithiasis and to identify candidate proteins for the treatment of kidney stones.

## 1. Introduction

Kidney stones are mineral deposits from renal papillae, and 80% of stones are calcium stones composed of calcium oxalate (CaOx) mixed with calcium phosphate [1]. Nephrolithiasis is a frequent chronic urological disease. The incidence and prevalence of kidney stones consistently increased in the past 3–4 decades globally, while the costs associated with stone disease have also increased [2]. In a prospective analysis, 67% of first-time symptomatic stone formers had stone recurrence at 5 years [3]. In China, the prevalence was 6.5% in men and 5.1% in women [4]. Meanwhile, the prevalence increased with age [5]. Patients with stones are at risk of hypertension, chronic kidney disease, and end-stage renal disease, resulting in heavy economic and social burden [6, 7]. To reduce the prevalence and recurrence rate of kidney stones, it is urgently needed to have a better understanding of the underlying mechanisms involved in nephrolithiasis based on high-throughput biotechnology.

High-throughput biotechnologies have enabled the collection of omics datasets to unearth the pathogenesis, biomarkers, and therapeutic targets of many diseases. Proteomics analysis has been applied to identify protein components in kidney stones and urine samples from patients with urolithiasis [8–10]. Researchers found that albumin and immunoglobulins were the most expressed proteins in the urine of urolithiasis patients [11], and the ratio of albumin to unidentified p24 proteins was higher in the urine of urolithiasis patients compared with controls [12]. Many proteins in CaOx stone samples were found to be significant, and they are involved in the inflammatory process and cell injury [13–16]. However, proteomics data on the kidney tissue of nephrolithiasis patients is relatively limited to date.

In this study, we performed iTRAQ/LC–MS/MS-based technology to investigate differentially expressed proteins in the kidney tissue of urolithiasis rats compared with controls. These results may help to characterize the mechanism of nephrolithiasis pathogenesis and to identify potential targets that interrupt nephrolithiasis development.

## 2. Methods

### 2.1. Animals and Kidney Stone Model

Adult male Sprague-Dawley (SD) rats weighing 250–300 g were supplied by the Laboratory Animal Center of Central South University (Changsha, China) and were housed in a controlled room with free access to food and water, where the 12-hour light-dark cycles temperature (22 ± 0.5°C) and humidity (40%-60%) were kept constant. All the experimental protocols were approved by the Ethics Committee for Animal Research of Central South University. The model of kidney stone rat was established as described previously [17]. Briefly, 30 rats were randomly divided into two groups. The control group rats were given normal drinking water *ad libitum* for 28 days, and the nephrolithiasis group rats were given 1% ethylene glycol (EG) (Sigma-Aldrich, Buchs, Switzerland) containing drinking water *ad libitum* for 28 days. Rats that became sick and stopped eating before 28 days were euthanized via cervical dislocation under intraperitoneal injection of ketamine (60 mg/kg) anesthesia.

### 2.2. Histopathological Study

Rats were anesthetized under sevoflurane, and blood was collected from the postcava in a no heparinized centrifuge tube and centrifuged at 3500 rpm for 15 min in separate serum. Then, rats were euthanized by exsanguinating, and the kidneys were removed. One kidney of each rat was fixed in 4% paraformaldehyde, dehydrated in ethanol solution, embedded in paraffin blocks, cut into 5-*μ*m serial slices, and stained for hematoxylin and eosin (H&E). Five sections per rat were selected randomly, and a polarizing microscope was used to evaluate the aggregation of CaOx crystals by the microscopic field with a magnification of 10∗40. The other kidney of each rat was snap-frozen in liquid nitrogen and stored at -80°C.

### 2.3. Preparation of Kidney Tissue Proteins

Total protein was extracted from each frozen kidney using a tissue protein extraction kit. Briefly, tissues were lysed by sodium dodecyl sulfate (SDS) lysis buffer containing a 1: 100 dilution of phenylmethanesulfonyl fluoride (Beyotime, Shanghai, China) in ice for 10 min. Then, the tissues were sonicated into ice and centrifuged at 12000 g for 10 min at 4°C. The concentrations of the extracted proteins were quantified using a bicinchonininc acid (BCA) protein assay kit (Beyotime, Shanghai, China). Protein samples (10 *μ*g) were separated by electrophoresis in a 12% SDS-PAGE gel and stained with Coomassie Brilliant Blue. Other proteins were stored at -80°C.

### 2.4. Protein Digestion and iTRAQ Labeling

The filter-aided sample preparation (FASP) procedure was used for protein digestion. Briefly, 100 *μ*g of protein was added to 120 *μ*l of reducing buffer (10 mM DTT, 8 M urea, 100 mM TEAB, pH 8.0) in 10-Kd ultrafiltration tubes. The mixture was incubated at 60°C for 1 h, IAA was added to the sample at a final concentration of 50 mM and incubated at room temperature for 40 min in darkness. Then, the tube was centrifuged at 4°C, and the filtrate was discarded. The sample was further added to 100 *μ*l 300 mM TEAB washing buffer and centrifuged at 12000 g for 20 min. This step was repeated twice. The filter unit was transferred to a new collection tube; next, the sample was added to 100 *μ*l 300 mM TEBA buffer and 2 *μ*l sequencing-grade trypsin (1 *μ*g/*μ*l, Hualishi, Beijing, China). The tube was left to digest at 37°C for 12 h and centrifuged at 12000 g for 20 min. Finally, the tube was added to 50 *μ*l 200 mM TEAB and centrifuged at 12000 g for 20 min, and the filtrates were collected and freeze-dried.

The lyophilized sample was resuspended in 100 *μ*l 200 mM TEAB with full vortexing, and 40 *μ*l per sample was used for iTRAQ labeling. iTRAQ labeling reagent (AB Sciex, CA, USA) was added to 200 *μ*l isopropanol at room temperature. The reagent was oscillated to mix fully for centrifugation, and this step was repeated once. Then, each sample was added to 100 *μ*L iTRAQ label reagent and kept at room temperature for 2 h. Last, the reaction was ended by adding 200 *μ*l HPLC water (Thermo Fisher Scientific, MA, USA) by incubating for 30 min. The labeling peptides were freeze-dried and stored at -80°C.

### 2.5. Peptide Fractionation Using Reversed-Phase Liquid Chromatography (RPLC)

iTRAQ-labeled peptides were separated on an Agilent 1100 HPLC system with an Agilent Zorbax Extend reversed-phase column (Agilent, Waldbronn, Germany). Buffer A (2% acetonitrile in HPLC water, Thermo Fisher Scientific, MA, USA) and buffer B (98% acetonitrile in HPLC water, Thermo Fisher Scientific, MA, USA) were used for fractionation. The flow rate was maintained at 300 *μ*L/min, and eluting peptides were monitored from 210 nm to 280 nm. The gradient program was set as follows: 0 ~ 8 min, 98% buffer A; 8.00~8.01 min, 98% ~95% buffer A; 8.01~38 min, 95% ~75% buffer A; 38~50 min, 75~60% buffer A; 50~50.01 min, 60~10% buffer A; 50.01~60 min, 10% buffer A; 60~60.01 min, 10~98% buffer A; 60.01~65 min, 98% buffer A. Samples were harvested from 8 min to 60 min, and fractions were collected over one-minute intervals and numbered from 1 to 15 with pipeline. The collected fractions were lyophilized for MS detection.

### 2.6. Nano LC-Electrospray Ionization (ESI)-MS/MS Analysis

Experiments were conducted on a Q Exactive mass spectrometer (MS) equipped with an Easy nano LC 1200 system (Thermo Fisher Scientific, MA, USA). The peptide was loaded into a C18 trap column (100-*μ*m ∗2-cm, 5-*μ*m particles, 100-Å aperture, Thermo Fisher Scientific, MA, USA) and separated by a C18 column (75-*μ*m∗50-cm, 2-*μ*m particles, 100-Å aperture, Thermo Fisher Scientific, MA, USA) at a flow rate of 300 nl/mim. Buffer A (0.1% formic acid) and buffer B (80% acetonitrile, 0.1% formic acid) were used in this step. The linear gradient consisted of 0 ~ 40 min, 5-30% buffer B, 40~54 min, 30-50% buffer B, 54~55 min, 50-100% buffer B, 55~60 min, and 100% buffer B.

MS was operated in positive ion mode, and the normalized collision energy was 30 eV. MS data were collected by dynamically selecting the top 10 most expressed precursor ions in the survey scan (300-1600 m/z) for higher-energy collisional dissociation (HCD) fragmentation. Survey scans were obtained at a resolution of 70,000 in m/z 200, the automatic gain control target was 1e6, and the maximum injection time was 50 ms. The HCD resolution spectra were 17,500 in m/z 200, the automatic gain control target value was 2e6, and the MS was dynamically excluded for 30 s.

### 2.7. MS Data Processing and Analysis

MS/MS spectra were searched by Q Exactive engine (Thermo Fisher Scientific, MA, USA) embedded into Proteome Discoverer 2.2 (Thermo Fisher Scientific, MA, USA) against the UniProt *Rattus norvegicus* database. The options used to identify proteins were as follows: peptide mass tolerance =20 ppm, MS/MS tolerance =0.1 Da, enzyme = Trypsin, missed cleavage = 2, fixed modification: carbamidomethyl (C), iTRAQ8plex (K), iTRAQ8plex (N-term), variable modification: oxidation (M), FDR ≤ 0.01. *p* ≤ 0.05 and fold-changes lower than 0.8 or higher than 1.2 were considered to be differentially expressed proteins.

### 2.8. Bioinformatics Analysis

Gene Ontology (GO), Kyoto Encyclopedia of Genes and Genomes (KEGG) analysis, and PPI networks were performed using the omics data analysis tool OmicsBean (http://www.omicsbean.com: 88). GO annotation includes biological process (BP), cellular component (CC), and molecular function (MF). KEGG analysis was performed to detect pathway clusters associated with differentially expressed proteins.

### 2.9. Parallel Reaction Monitoring (PRM) for Target Protein Validation

The extraction and digestion of protein were conducted in the same manner as the iTRAQ experiment. Then, the peptides were loaded into a C18 trap column for desalting before RPLC in an Agilent 1100 HPLC system (Agilent, Waldbronn, Germany). Linear gradients with acetonitrile ranging from 2% to 90% over 65 min were used. Twenty-eight proteins, including eight reference proteins, were selected for validation by PRM on a Q Exactive MS (Thermo Fisher Scientific, MA, USA). The instrument was worked in the positive ion mode, and the normalized collision energy was 27 eV. A survey scan (350-1000 m/z) was obtained at a resolution of 120000, an automatic gain control target of 3e6, and the maximum injection time of 100 ms. The fragments were detected at a resolution of 30000, an automatic gain control target of 2e5, a maximum injection time of 80 ms, and a dynamically excluded MS of 40 s. The results were analyzed by Skyline (AB Sciex, CA, USA). Quantification results were normalized to the standard reference.

### 2.10. Statistical Analysis

All statistical data were processed with GraphPad Prism software version 6 (GraphPad, CA, USA), and the data were analyzed by Student's t-test (two-tailed and unpaired) and expressed as the mean ± standard deviation (SD). A *p* value <0.05 was considered to be statistically significant.

## 3. Results

### 3.1. Histopathological Changes in Kidney Tissue

**Figure 1:**
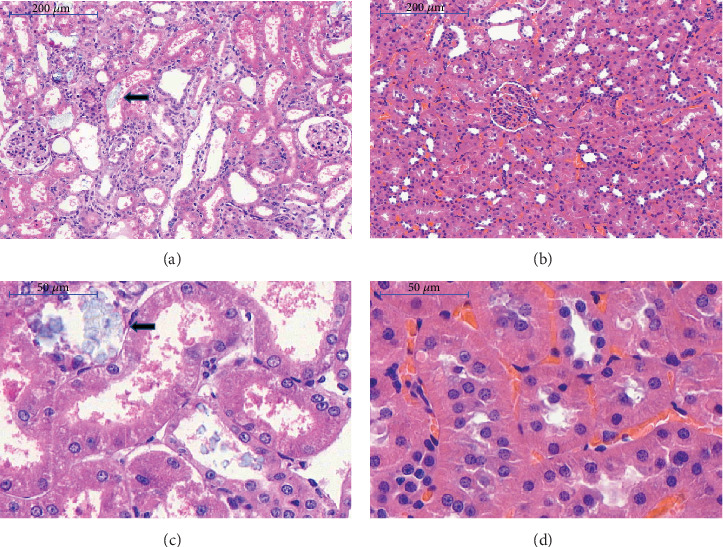
Histopathological changes in the kidney of rats after a 28-day administration of 1% EG or saline. Black arrows in a and c indicate the deposition of a large amount of calcium oxalate crystal in the nephrolithiasis group; control group showing the normal renal tubule (b, d). Microscopic images are representative of n = 4 in each group.

Four rats in the nephrolithiasis group were likely to die of kidney failure, and 26 were included in the study. H&E staining (Figure 1(a), 1(c)) demonstrated that 1% EG administration induced expressed crystal calcium oxalate crystal deposition with high refractivity (black arrow). The structure of the renal parenchyma was destroyed by CaOx crystals from the nephrolithiasis group. In the control group, no CaOx crystals were formed, and the renal parenchyma structure remained intact (Figure 1(b), 1(d)).

### 3.2. Identification of Differentially Expressed Proteins (DEPs)

**Figure 2:**
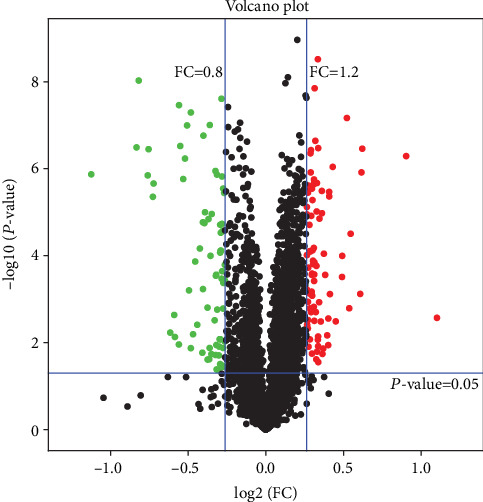
Volcano plot of differentially expressed proteins in kidney tissues. All proteins were plotted with log_2_ fold change on the *x*-axis and − log_10_ (*P* value) on the *y*-axis. The red dots in the upper right (ratio> 1.2) and the green dots upper left (ratio< 0.8) sections with *P* < 0.05 represent proteins that were significantly up and down regulated between the two group. Black dots are proteins that were the same in the two compared groups.

**Figure 3:**
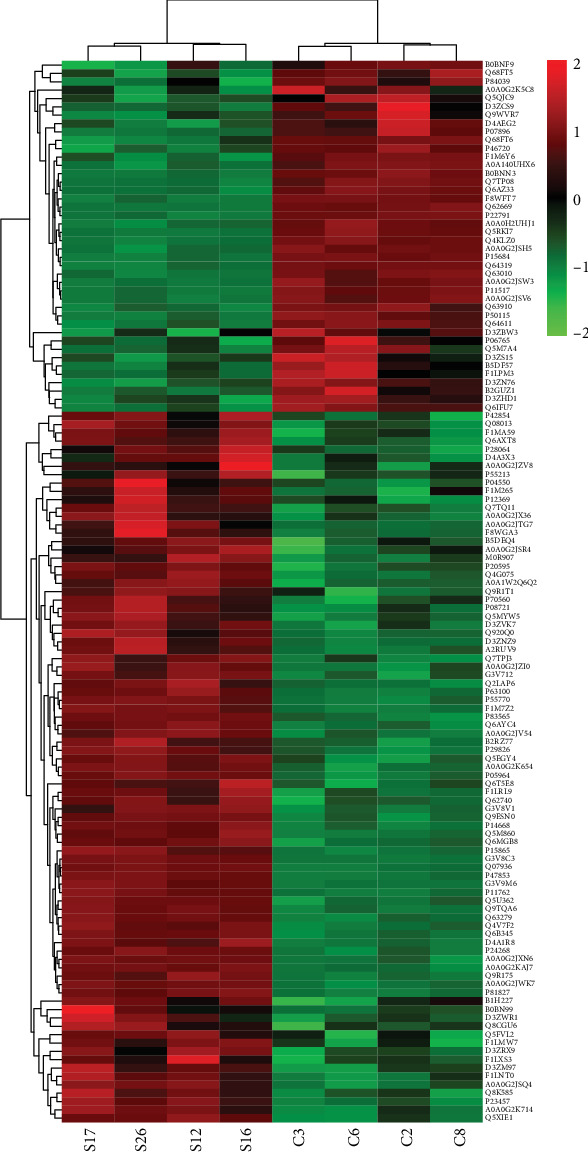
Hierarchical cluster analysis of the differentially expressed proteins. Each column represents a tissue sample and each line represents a differentially expressed protein. The color scale going from green (low) to red (high) indicates the expression levels of DEPs. Red and green indicate up- and down-regulation, respectively. S represents nephrolithiasis group rats and C represents control rats.

A total of 3495 proteins were identified in the kidneys of nephrolithiasis rats and control rats matched in the UniProt *Rattus norvegicus* database (Supplementary Table 1). Proteins with fold change (FC) > 1.2 or< 0.8 with more than two peptides were regarded as differentially expressed proteins (*p* < 0.05). Among the 3495 identified proteins, 127 significantly differentially expressed proteins (85 were upregulated and 42 were downregulated) were found in nephrolithiasis rats compared with the controls (Supplementary Table 2). Serpinb1b (fold change = 2.15) and Amacr (fold change = 0.46) were the most significantly up- and downregulated differentially expressed proteins in nephrolithiasis rats compared with controls. Statistical results of protein quantification are shown in the volcano plot (Figure 2). Visualization by hierarchical clustering analysis of candidate proteins showed clear differences in kidneys between kidney stone rats and controls (Figure 3).

### 3.3. Gene Ontology Analysis

**Figure 4:**
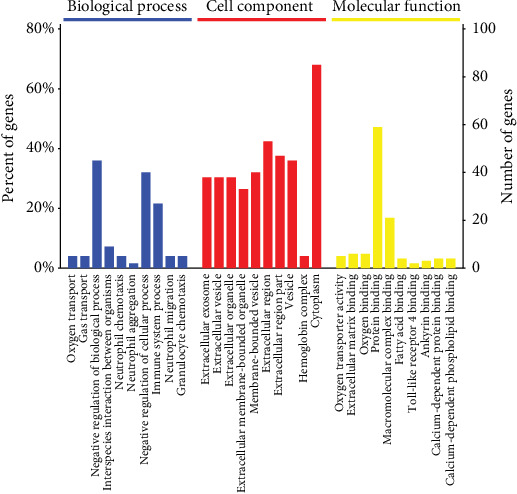
GO analysis of the 127 differential expressed proteins for functional classification. Blue, red and yellow bars represent biological processes, cellular components, and molecular functions, respectively. Terms in the same category were ranked based on the *P*-values. The ordinates on the right and left represent the number of DEPs in each entry and their percentage in the total number of differential proteins.

Gene Ontology (GO) functional annotation was used to uncover the functional classification of 127 identified DEPs based on biological process (BP), molecular function (MF), and cellular component (CC) categories. GO enrichment analysis showed that the DEPs were mainly related to CC. BP analysis showed that the majority of the proteins were primarily involved in oxygen transport, gas transport, negative regulation of biological processes, interspecies interaction between organisms, and neutrophil chemotaxis. According to CC analysis, DEPs were clearly enriched in extracellular exosomes, extracellular vesicles, extracellular organelles, extracellular membrane-bounded organelles, and membrane-bounded vesicles. MF showed DEPs were primarily enriched in oxygen transporter activity, extracellular matrix binding, oxygen binding, protein binding, and macromolecular complex binding (Figure 4).

### 3.4. Kyoto Encyclopedia of Genes and Genomes (KEGG) Pathway Analysis

**Figure 5:**
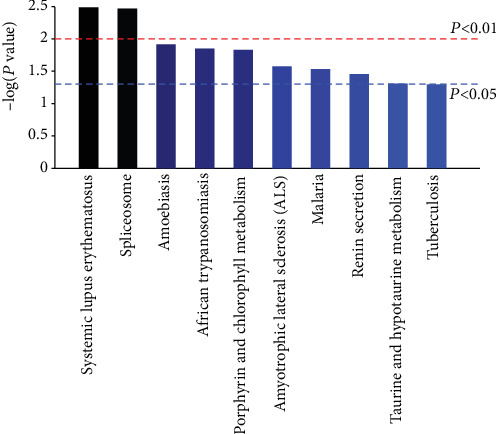
KEGG pathway enrichment analysis of differentially expressed proteins with the ten highest enrichment scores. The x-axis shows the pathways. The y-axis colored with gradient color is -log (*P* value) showing the enrichment score, the bigger the enrichment score, the smaller the *P* value, indicating that the enrichment of DEPs in given pathways was significant.

KEGG pathway enrichment analysis was conducted to identify the functions of DEPs. The results showed that DEPs in nephrolithiasis rats compared with the control rats were involved in 117 KEGG pathways. As shown in Figure 5, the top ten significant pathways were as follows: systemic lupus erythematosus, spliceosomes, amoebiasis, African trypanosomiasis, porphyrin and chlorophyll metabolism, Amyotrophic lateral sclerosis (ALS), Malaria, Renin secretion, Taurine and hypotaurine metabolism, and Tuberculosis. The systemic lupus erythematosus pathway was the most representative pathway, including 4 DEPs, followed by the spliceosome and amoebiasis pathways, encompassing 4 and 3 DEPs, respectively.

### 3.5. Protein–Protein Interaction (PPI) Network of Differentially Expressed Proteins

**Figure 6:**
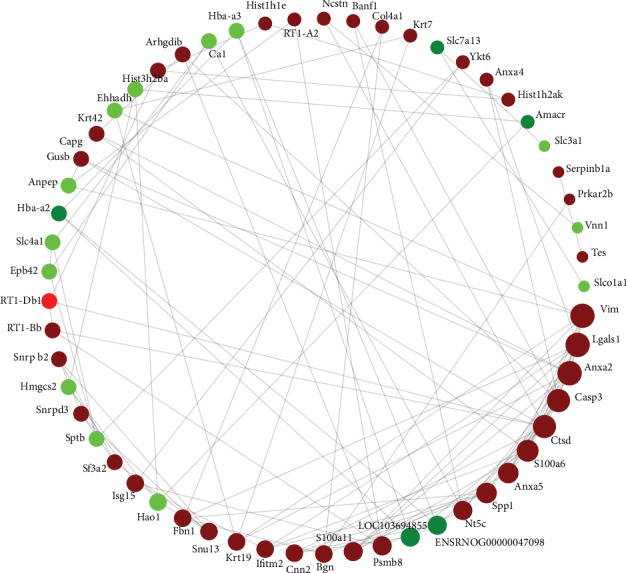
Protein-protein interaction networks of the identified differentially expressed proteins. PPI analysis was based on fold changes of protein expression. Red dots and green dots represent up regulated and down regulated proteins, respectively, the gray colored lines represent interactions between proteins and proteins.

To further uncover the regulatory role of DEPs in nephrolithiasis rats compared with the controls, interactions between any set of two DEPs in this study were used to build a regulatory network using OmicsBean (Figure 6). The results demonstrated that the DEPs constructed a complicated regulatory network containing 57 nodes and 102 edges. Vim and lgals1 were the most important hubs interacting with 9 proteins.

### 3.6. Differentially Expressed Proteins Validated by PRM

**Figure 7:**
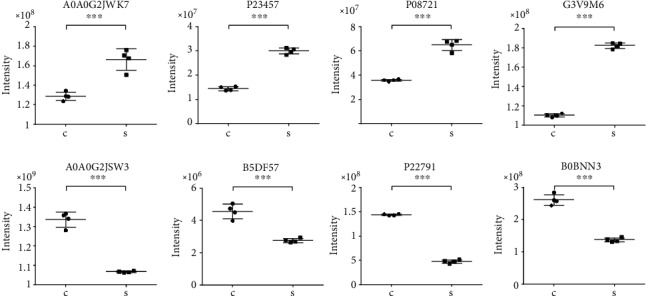
Relative expression levels of selected proteins measured by PRM in kidney tissues of nephrolithiasis rats and control rats. Values are presented as mean ± SD (n = 4 in each group). ∗*P* < 0.05, ∗∗*P* < 0.01, ∗∗∗*P* < 0.001. S represents nephrolithiasis rats, and C represents matched control rats.

The protein expression levels of 8 differentially expressed proteins of great interest were selected for quantification by PRM to confirm the iTRAQ results. All target proteins have unique peptides. As shown in Figure 7, the changing trend of the results detected by PRM was largely consistent with the results observed by iTRAQ. Four proteins (A0A0G2JWK7, P23457, P08721, and G3V9M6, with the gene names Tagln, Akr1c9, Spp1, and Fbn1, respectively) were upregulated, and four proteins (A0A0G2JSW3, B5DF57, P22791, and B0BNN3, with the gene names Hbb, Epb42, Hmgcs2, and Ca1, respectively) were downregulated in nephrolithiasis rats compared with the control rats. However, due to the different detection methods, the specific values of the results were not the same. Thus, the results obtained by iTRAQ were reliable.

## 4. Discussion

Urolithiasis is a worldwide problem, and the formation of stones in the kidney stems from many underlying disorders but has not been thoroughly elucidated to date [18]. In our previous study, oral medication of 1% ethylene glycol with drinking water from SD rats for 4 weeks induced CaOx, kidney stones, and renal hypofunction [17]. In the present study, we used the same method to induce rat models into urolithiasis successfully. High-throughput biotechnologies have assisted in exploring the underlying mechanisms related to the development of various diseases [19]. It is imperative to further study the molecular mechanisms of urolithiasis based on advances in proteomics. We performed iTRAQ combined with LC-MS/MS to obtain proteome profiles of the kidneys of nephrolithiasis and control rats.

We identified 127 significantly DEPs (85 upregulated and 42 downregulated) in kidney tissues of nephrolithiasis rats compared with controls. Then, we studied the characterization of altered proteins in depth. The most strongly upregulated protein, Serpinb1b, was one of the most efficient inhibitors of the neutrophil granule proteases and played an important role in modulating oxidative stress [20]. The most strongly downregulated protein, Amacr, involved in the metabolism of branched chain fatty acids, was an immunomarker for prostate cancer and papillary renal cell carcinoma [21]. A researcher reported a patient with kidney dysfunction secondary to nephrolithiasis and found AMACR-positive renal adenomatosis accompanied by papillary carcinoma in a nephrectomy specimen [22]. The expression levels of 8 DEPs, including four upregulated proteins (Tagln, Akr1c9, Spp1, and Fbn1) and four downregulated proteins (Hbb, Epb42, Hmgcs2, and Ca1), were verified by PRM, the results of which were in keeping with the iTRAQ results. Bioinformatics analyses, including GO, KEGG, and PPI networks, were conducted to predict the potential function of DEPs in kidney stone rats.

Thus, our results provide an integrative understanding of the functions of DEPs in EG-induced kidney stone rats, and our findings could help to obtain novel prophylactic and therapeutic measures for kidney stones.

GO analysis revealed that the majority of the DEPs were primarily involved in oxygen transport, gas transport, negative regulation of biological processes in BP, extracellular exosome, extracellular vesicle, extracellular organelle in CC, and oxygen transporter activity, extracellular matrix binding, and oxygen binding, in MF. Oxygen transport is important to preserve the proper matching of oxygen supply to demands from many tissues. Tissue hypoxia is now recognized as a unifying pathway in chronic kidney disease. In diabetic and hypertensive kidneys, sustained hyperglycemia and hypertension increased oxygen consumption and the levels of oxidative stress, leading to renal damage and function reduction [23]. In addition, the medulla was at high active consumption rate, medullary oxygenation was hypersensitive to blood flow, and tissues were vulnerable to hypoxic injury [24, 25]. Calcium oxalates crystal binding and oxalate-induced oxidative stress in renal epithelial cells. A previous study showed that calcium oxalate crystal deposition induced oxidative stress in human primary renal epithelial cells [26]. Secretory protein SPP1 was involved in extracellular matrix binding of MF. Spp1, also known as osteopontin (OPN), is a significant component of the calcium oxalate crystal matrix and plays a vital role in modulating stone formation [27–29]; however, its role in the formation in kidney stones remains controversial. Some researchers found OPN to be a powerful synergistic inhibitor of renal calcinosis and stone formation [30, 31]. Other studies found that OPN attributed to renal crystal formation *in vitro* and *in vivo* [32–35] by its characteristic structure, including two calcium-binding sites and Arg-Gly-Asp (RGD) sequences. Bhardwaj R found calcium oxalate crystal deposition was associated with the expression of OPN and endoplasmic reticulum stress [36]. In the 1% EG-induced rat kidney stone model, the expression level of OPN was increased, accompanied by mitochondrial collapse, oxidative stress, and activation of the apoptotic pathway, resulting in the initial process of renal calcium crystallization [37].

KEGG pathway analysis also revealed important insights into kidney stones. Systemic lupus erythematosus and spliceosome, amoebiasis pathways were found to be enriched in kidney stones. Renal damage is common in systemic lupus erythematosus, and the most severe form is diffuse proliferative lupus nephritis [38]. Oxalate stress induced the nuclear membrane damage and increased the expression of nuclear pore complex oxalate binding protein p62 at the same time. p62 autoantibodies were observed in hyperoxaluria patients and systemic lupus erythematosus patients [39]. Renal tubular acidosis (RTA) is a rare complication in systemic lupus erythematosus. Patients with systemic lupus erythematosus accompanied by type 1 RTA often have hypokalemia and normal glomerular filtration rates and a tendency to develop nephrocalcinosi and renal calculi [40].

The PPI network showed that Vim was the most vital hubs interacting with 9 proteins and Anxa 2 was interacted with 8 proteins. Vim and Anxa 2 were upregulated in the kidney tissue of CaOx kidney stones rats in the present study. Vim is an intermediate filament expressed mainly in mesenchymal cells. Researchers found that hyperoxaluria caused upregulation of Vim, increased Vim immunoreactivity, and induced tubular epithelial cells in osteoblast-like cells in hydroxy-l-proline-induced CaOx kidney stone rats [41]. Anxa 2 was the most prominent calcium oxalate monohydrate crystal-binding protein. Anxa 2 could regulate the adhesion of calcium oxalate monohydrate crystals to renal epithelial cells, and altered exposure of Anxa 2 on the surface of renal tubular epithelial cells could promote kidney stone formation [42].

In the present study, we identified 127 DEPs and potential signaling pathways in EG-induced kidney stone rat models based on iTRAQ. PRM validated the differential expression levels of Tagln, Akr1c9, Spp1, Fbn1, Hbb, Epb42, Hmgcs2, and Ca1. These DEPs might be valuable clinical markers contributing to disease onset and progression or novel prevention and treatment targets for alleviating nephrolithiasis.

## 5. Conclusions

This study's proteomics data presented a comprehensive perspective on the potential biological functions of DEPs in the mechanisms governing nephrolithiasis. This study's results may help to identify possible targets for the prophylactic and therapeutic measurement of kidney stones, and more research is needed to thoroughly elucidate this subject.

## Data Availability

The datasets used and/or analyzed during the current study are available from the corresponding author on reasonable request.

## Abbreviations

iTRAQ: Isobaric tags for relative and absolute quantification
DEPs: Differentially expressed proteins
KEGG: Kyoto encyclopedia of genes and genomes
PPI: Protein–protein interaction
PRM: Parallel reaction monitoring
CaOx: Calcium oxalate
EG: Ethylene glycol
H&E: Hematoxylin and eosin
RIPA: Radio Immunoprecipitation assay
BCA: Bicinchonininc acid
SDS-PAGE: Sodium dodecyl sulfate–polyacrylamide gel electrophoresis
DTT: Dithiothreitol
TEAB: Triethylamonium bicarbonat
IAA: Iodoacetamide
RPLC: Reversed-phase liquid chromatography
HPLC: High performance liquid chromatography
MS: Mass spectrometers
HCD: Higher-energy collisional dissociation
GO: Gene ontology
KEGG: Kyoto encyclopedia of genes and genomes
PRM: Parallel reaction monitoring
OPN: Osteopontin
RGD: Arg-Gly-asp
RTA: Renal tubular acidosis.

## Supplementary Materials

Supplementary 1 Supplementary table 1 Identified proteins in the kidney of nephrolithiasis rats and the control rats.

Supplementary 2 Supplementary table 2. Differentially expressed proteins in the kidney of nephrolithiasis rats and the control rats.
